# Traumatic bilateral fourth nerve palsy: Double vision induced by downward gaze after head injury

**DOI:** 10.1002/jgf2.310

**Published:** 2020-03-11

**Authors:** Masaki Tago, Yoshio Hisata, Risa Hirata, Mika Sakaguchi, Naoko E Katsuki, Shu‐ichi Yamashita

**Affiliations:** ^1^ Department of General Medicine Saga University Hospital Saga Japan; ^2^ Department of Ophthalmology Faculty of Medicine Saga University Saga Japan

**Keywords:** concussion, double vision, fourth nerve palsy, head injury, trauma

## Abstract

Clinicians should be alert to traumatic fourth nerve palsy in patients with double vision after head injury. In most cases with traumatic fourth nerve palsy, the imaging tests fail to show abnormal findings. In the cases of bilateral superior oblique paresis, the sensitivity of Bielschowsky head‐tilt test is 40%.

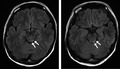

A 32‐year‐old woman with no past medical or family history was transported to our hospital by ambulance because of a loss of consciousness without convulsions or incontinence. She had been taking a bath after drinking alcohol (Day 0). Although her level of consciousness improved after a few minutes, she had no memory of events before and after the loss of consciousness. Upon admission, she was awake and alert. Her body temperature was 36.1°C, pulse was 78 beats/min, blood pressure was 82/50 mm Hg, respiration was 20 breaths/min, and oxygen saturation was 99% on room air. Physical examination showed no significant abnormalities other than palpebral subcutaneous hematoma on the left side and tenderness over the occipital area of her head. Because laboratory tests, electrocardiography, chest x‐ray, computed tomography, and electroencephalography also failed to reveal abnormalities, she was diagnosed with a concussion resulting from banging her head in the fall due to neurally mediated syncope.

On Day 1, she developed double vision induced by downward gaze. Ophthalmologic examination revealed restriction of eye movements in both eyes when looking in the inferomedial direction, with negative Bielschowsky head‐tilt test (Figure 1). Because there was no traumatic change in the pupil and retina, superior oblique muscle paresis due to traumatic bilateral fourth nerve palsy was suspected. T2‐weighted brain magnetic resonance imaging (MRI) on Day 2 revealed an area of slightly high attenuation around the left inferior colliculus of the midbrain (Figure 2). We finally made a diagnosis of traumatic bilateral fourth nerve palsy. Although her diplopia improved to some degree with conservative treatment, mild bilateral diplopia remained. That was corrected with prism glasses.

**FIGURE 1:**
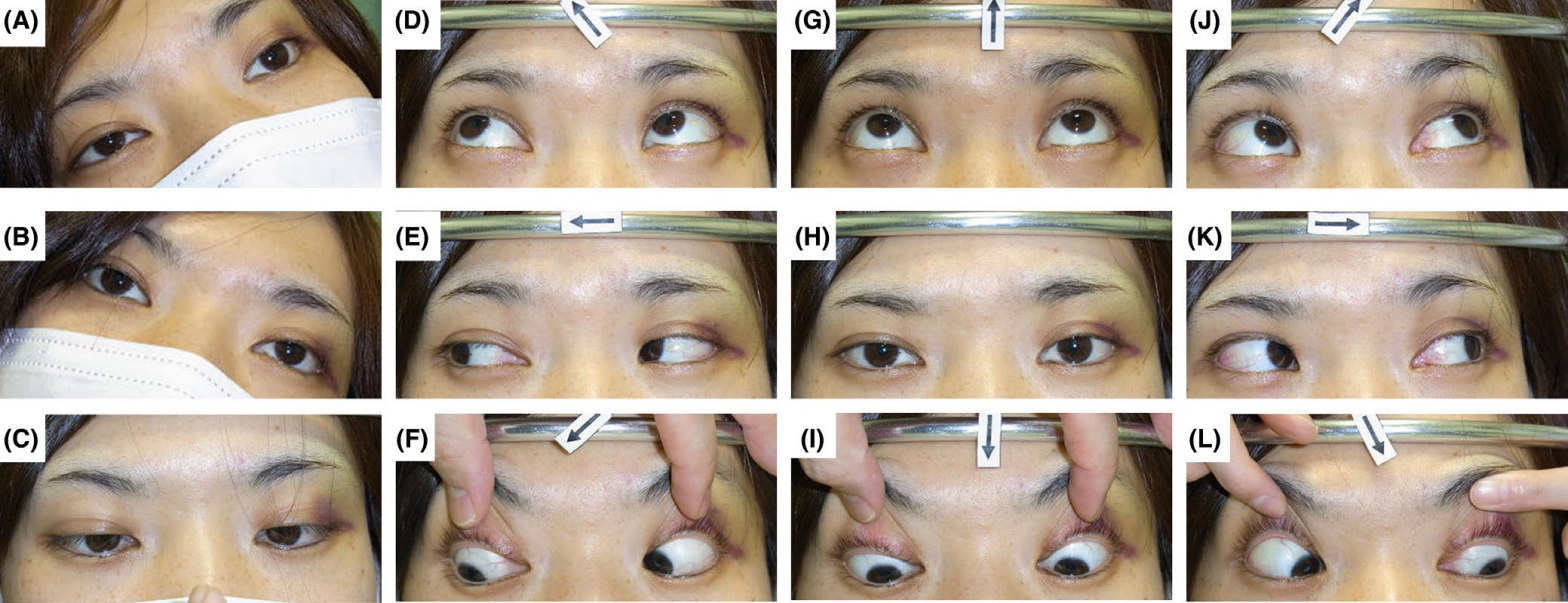
The findings of the Bielschowsky head‐tilt test and eye movements. (A, B) Bielschowsky head‐tilt test was negative, which means that eye position was normal on bilateral head tilt. (F, L) Restriction of eye movements was observed in both eyes when looking in the inferomedial direction. (C‐E, G‐K) The eye movements in all other directions were normal

**FIGURE 2:**
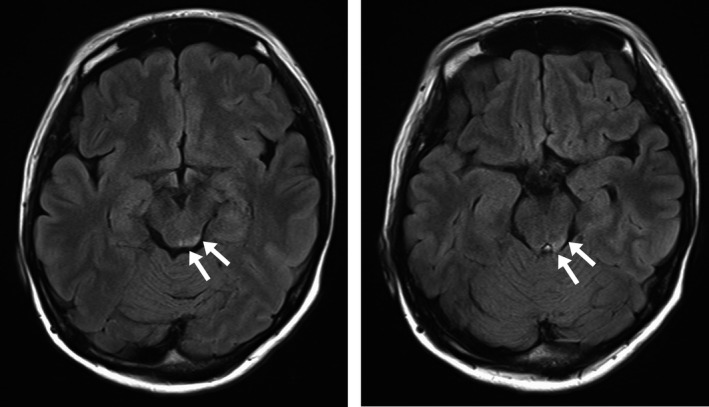
Brain magnetic resonance imaging. T2‐weighted images revealed slightly high attenuation around the inferior colliculus of the midbrain on the left side (arrows)

The most common cause of fourth nerve palsy is congenital anomaly, followed by trauma, microvascular disease, and idiopathic diseases.^1^, ^2^ Although bilateral involvement occurs in only 10.7% of all fourth nerve palsies, 20.7% of traumatic cases have bilateral involvement.^1^ The present case showed marked restriction of eye movements in both eyes only when looking in the inferomedial direction, due to paresis in the bilateral superior oblique muscles. There were no abnormalities in eye movements in horizontal or upward gaze caused by reversing hypertropia or overactivity of the inferior oblique muscles.^3^ Furthermore, the Bielschowsky head‐tilt test, whose sensitivity in cases of bilateral superior oblique muscle paresis is reportedly 40%, was negative, making it rather difficult to make a rapid and correct diagnosis at the bedside.^4^

The fourth nerve decussates within the lower dorsal midbrain at the level of the inferior colliculus, before emerging on the contralateral side of the brainstem. Therefore, if the fourth nerve suffers injury at the decussation, isolated bilateral fourth nerve palsies could appear.^5^ Regrettably, in most cases of traumatic fourth nerve palsy, the imaging tests failed to show abnormal findings. Only a few cases have been reported in which lesions in or around the dorsal midbrain, suggesting hemorrhage, have been detected through imaging studies; those benefited from MRI of the brain as in our case or from computed tomography.^5^

When seeing a patient with double vision after head injury, and no apparent abnormalities in the pupil or retina, traumatic fourth nerve palsy due to midbrain lesions should be ruled out with timely and careful neurological examination and brain imaging tests, especially MRI.

## CONFLICT OF INTERESTS

The authors have stated explicitly that there are no conflicts of interest in connection with this article.

## INFORMED CONSENT

The patient was given informed consent, and patient anonymity was preserved.
