# Corrigendum

**DOI:** 10.1002/jgf2.371

**Published:** 2020-11-25

The authors would like to draw the reader's attention to errors in the manuscript, entitled 'Traumatic bilateral fourth nerve palsy: Double vision induced by downward gaze after head injury' which was published in *Journal of General and Family Medicine*, Vol 21, Issue 4.^1^

The below text in the paragraph “On Day 1, she developed…with prism glasses.” is to be corrected:

T2‐weighted brain magnetic resonance imaging (MRI) on Day 2 revealed an area of slightly high attenuation around the left inferior colliculus of the midbrain (Figure 2).

The correct text is as follow:

Brain magnetic resonance imaging (MRI) on Day 2 revealed a slightly high‐intensity area around the left inferior colliculus of the midbrain on fluid‐attenuated inversion recovery images (Figure 2).

The correct Figure 2 legend is as follow:

Brain magnetic resonance imaging. Fluid‐attenuated inversion recovery images revealed a slightly high‐intensity area around the inferior colliculus of the midbrain on the left side (arrows)

The authors apologize for the errors and any confusion they may have caused.
